# *In vitro* chemosensitivity of a canine tumor venereal transmissible cancer cell line

**DOI:** 10.3389/fvets.2022.972185

**Published:** 2022-08-18

**Authors:** Moisés Armides Franco Molina, Edson Antonio Santamaría-Martínez, Silvia Elena Santana Krimskaya, Diana Ginette Zarate-Triviño, Jorge R. Kawas, Yareellys Ramos Zayas, Natanael Palacios Estrada, Heriberto Prado García, Paola Leonor García Coronado, Cristina Rodríguez Padilla

**Affiliations:** ^1^Laboratorio de Inmunología y Virología, Facultad de Ciencias Biológicas, Universidad Autónoma de Nuevo León, San Nicolás de los Garza, NL, Mexico; ^2^Posgrado Conjunto Agronomía-Veterinaria, Universidad Autónoma de Nuevo León, Escobedo, NL, Mexico; ^3^Laboratorio de Onco-Inmunobiologia, Departamento de Enfermedades Crónico-Degenerativas, Instituto Nacional de Enfermedades Respiratorias, Ciudad de Mexico, Mexico

**Keywords:** chemosensitivity, canine transmissible venereal tumor, cancer cell line, chemotherapy, vincristine

## Abstract

The canine transmissible venereal tumor (CTVT) is the most common malignity in dogs. Because there are reports that this tumor is resistant to vincristine sulfate, the chemotherapeutic options are scarce, and the development of new therapeutic approaches is necessary. In this study, we evaluated the cytotoxic activity of vincristine, doxorubicin, temozolomide, panobinostat, toceranib, gemcitabine, cisplatin, fluorouracil, cyclophosphamide, and methotrexate on a CTVT cell line, determining that all drugs decreased the viability in a dose-dependent manner. Furthermore, they inhibit cellular migration in a time- and drug-dependent manner, as evaluated by the wound healing assay. On the other hand, vincristine, panobinostat, gemcitabine, toceranib, cyclophosphamide, and methotrexate increased the percentage of cells in the subG1 phase, and doxorubicin, temozolomide, gemcitabine, toceranib, and methotrexate decreased the percentage of cells in the synthesis phase. To efficientize the use of vincristine, only toceranib increased the cytotoxic effect of vincristine in a synergistic manner. Our results confirm the use of vincristine as the gold standard for CTVT treatment as monotherapy and suggest the use of a combinatorial and sequential treatment with toceranib.

## Introduction

The canine transmissible venereal tumor (CTVT) is a neoplasia transmitted sexually by the transplantation of viable tumor cells; although it mostly occurs in the genital region, implantation can occur in any part of the body, especially if there are surface abrasions or loss of integrity of the skin (1, 2). Vincristine sulfate is a commonly used chemotherapeutic to treat this neoplasm, reporting a high degree of efficacious and complete remission in more than 90% of the cases. However, there are clinical reports that mention vincristine sulfate resistance in CTVT (3). The chemotherapeutic options to be used in the case of CTVT treatment or resistance are limited due to the scarce clinical and *in vitro* chemo sensibility studies that suggest new options; one of the reasons that for lack of studies is that no CTVT cell line exists except for the one used in this study (2). Today, studies in patients and *in vitro* experiments using cell cultures are used to analyze the response to drug treatments. The *in vitro* experiments are cheaper and used to suggest a first reflection of the *in vivo* situation for results translation to the clinical setting (4, 5).

The chemotherapeutic options used to treat cancer act by different mechanisms of action at different cellular levels, by altering or inhibiting cellular growth. Alkylating agents (cyclophosphamide, cisplatin, temozolomide) act by chemically altering cellular DNA (cyclophosphamide, cisplatin, temozolomide); anti-metabolic drugs (methotrexate and fluorouracil) work like the building blocks of DNA by mimicking the role of purine or pyrimidine in stopping cell division (methotrexate and fluorouracil). Alkaloids (vincristine sulfate) block cell division by inhibiting microtubule function (vincristine sulfate). Alkylating agent doxorubicin is non-specific in the cell-cycle stage because it binds with DNA and thus prevents RNA synthesis, a key step in the creation of proteins, which are necessary for cell survival. Multi-target receptor tyrosine kinase inhibitor (toceranib) disrupts several members of the split kinase RTK family, including vascular endothelial growth factor receptor 2 (VEGFR2), platelet-derived growth factor receptors-alpha and -beta (PDGFR α/β), KIT, and Flt-3, among others (6). Panobinostat, which is a non-selective histone deacetylase inhibitor acting over histone and non-histone proteins, results in gene transcription and protein activity changes (7). Gemcitabine is a nucleoside analog with two fluorine atoms replacing the hydroxyl group on the ribose; as a product, gemcitabine is transformed into its active metabolite that works by replacing the building blocks of nucleic acids during DNA elongation, arresting tumor growth, and promoting apoptosis of the malignant cells. For the performance of this study, we decided to use some of the most common chemotherapy drugs (described above) used in veterinary cancer medicine which, due to their mechanism of action, can affect different molecular levels that can eradicate CTVT cancer cells (antimetabolites, DNA alkylating agents, HDAC inhibitors, and RTKs inhibitors). *In vitro* determination of chemosensitivity serves as a baseline for further experimental approaches aiming to discover more chemotherapy drugs with potential clinical significance against CTVT. This study aimed to establish an *in vitro* tumor chemosensitivity assay to select the most appropriate chemotherapy option for CTVT patients by indicating resistance or sensitivity to drugs.

## Materials and methods

### Chemicals

Fetal bovine serum (FBS), and alamarBlue TM Cell Viability Reagent, were purchased from Thermo Fisher Scientific, USA; vincristine sulfate (1 mg/ml) (Pfizer), doxorubicin hydrochloride (Pfizer), temozolomide (Sigma–Aldrich, USA), panobinostat (Sigma–Aldrich, USA), toceranib (Sigma–Aldrich, USA), fluorouracil (50 mg/ml) (Pfizer), cyclophosphamide (500 mg, Sanfer), methotrexate (25 mg/ml, Pfizer), gemcitabine (1 mg, PISA, S.A de C.V.), and cisplatin (50 mg/50 ml, LEMERLY, S.A de C.V.). DMEM/F12 (Thermo Fisher Scientific, USA), and penicillin/streptomycin (Thermo Fisher Scientific, USA).

### CTVT cell line

For maintenance, CTVT cells stablished by our laboratory (2) were cultured in a 75 cm^2^ culture flask (Corning, USA) containing Dulbecco's modified Eagle's medium (DMEM/F-12) and 4-(2-hydroxyethyl)-1-piperazineethanesulphonic acid (HEPES) (Gibco; Thermo Fisher Scientific, USA) with 10% fetal bovine serum (FBS) (Gibco), as well as antibiotics and antimycotics (Antibiotic-Antimycotic 100X; Gibco), at 37 °C with 5% CO_2_. StemPro Accutase (Gibco) was used to detach the cells from the flask (2).

### Alamar blue assay

CTVT cells (5 × 10^3^ cells/well) were seeded on 96-well flat-bottom plates and incubated for 24 h at 37 °C in a 5% CO_2_ atmosphere. After incubation, the culture medium was removed, and chemotherapeutics diluted in the same medium were added at concentrations ranging from 0 to 100 μg/ml. The plates were then incubated for 24 h at 37 °C, and 5% CO_2_ atmosphere. Thereafter, the supernatant was removed, and cells were washed twice with DMEM/F-12 medium. Cell viability was determined by the Alamar blue method (8), and cytotoxicity was expressed as the concentration of 50% (CC_50_) cell growth inhibition. Results are presented as the mean ± SD from three independent experiments (with three replicates for each concentration per experiment). The CC_50_ of each drug was calculated from the viability data obtained with the Alamar blue assay using the Quest GraphTM ED50 Calculator program (AAT Bioquest, Inc.) (9, 10). Quest Graph™ ED50 Calculator (11, 12). Six replicates were included in the analysis.

### *In vitro* wound healing assay

CTVT cells (3 × 10^5^ cells/well) were seeded in 24-well plates to grow in a monolayer for 24 h. Then a sterile 20–200 μl pipette tip was held vertically to scratch across each well. The detached cells were removed by washing with 500 μl PBS and shaken at 500 rpm for 5 min. A total of 500 μl of fresh medium with or without diluted samples were added afterward and incubated for 72 h. Before image acquisition, the plate was washed with 500 μl pre-warmed PBS and gently shaken for 30 s. Then, a pre-warmed medium or sample was added again, and pictures were taken. The scratch closure was monitored and imaged using Axiovert 25 microscope (Zeiss, Germany). The analysis of images was performed using the Image J software (13) which calculates the scratch area (open wound area) for each image. Three independent experiments and presented as mean ± SD from three independent experiments (with three replicates for each concentration) (14).

### Clonogenic survival assays

CTVT single-cell suspensions of exponentially growing cultures were seeded into six-well plates at 1 × 10^5^ cells (expecting a resulting countable number of colonies per well) and allowed to adhere. Upon adherence, the cell culture medium was refreshed (2 ml/well in most experiments), and cells were treated with drugs at different doses of dilution. Cells were then incubated at 37 °C for 15 days. Fixation and staining were performed using 80% ethanol containing 8% methylene blue (Sigma Aldrich) morphology and colony size, counting was performed at 10- to 40-fold magnification. Results are presented as mean ± SD from three independent experiments (with three replicates for each concentration).

### Cell cycle analysis

After treatment, the cells were collected and washed with phosphate buffer (PBS) pH 7.2, fixed in 70% v/v ethanol, and stored at −20 °C. For cell cycle analysis, samples were washed with PBS and incubated in 0.5% v/v triton X-100, 7-amino actinomycin D (7-AAD) (10 μg/ml) solution under dark conditions at room temperature for 20 min. The DNA content was determined using a FACSCanto II flow cytometer (Becton Dickinson, San Jose, CA, USA). For cell cycle analysis and SubG0 peak evaluation, a total of 20,000 events from 7-AAD-area vs. 7-AAD-wide gate were acquired. The results were analyzed using FlowJo Software (Becton Dickinson).

### Drug combination effectivity analysis

The dose effects of each drug or drug combination were defined by the half-maximal inhibitory concentration (IC_50_) values and were calculated using the Chou-Talalay method (11). For each drug and drug combination IC_50_ was calculated, and the drug combination's synergistic or antagonistic effect was quantified. Drug combination effects were defined by the resulting combination index (CI) theorem of Chou-Talalay, which offers a quantitative definition for additive effects (CI = 1), synergism (CI < 1), and antagonism (CI > 1). The dose-reduction index (DRI) is defined by the DRI equation of Chou-Talalay, and it is a measure of how many folds the dose of each drug in a synergistic combination may be reduced at a given effect level when compared with the doses of each drug alone. The data were analyzed with CompuSyn software (10), and dose–response curves were constructed with GraphPad Prism 6.

### Statistical analysis

One-way analysis of variance (ANOVA) and Dunnett's or Tukey's *post hoc* test were used for data analysis. All the results were expressed as mean ± SD, and *p*-values < 0.05 were considered statistically significant.

## Results

### Single drugs decrease the viability of CTVT cells

**Figure 1:**
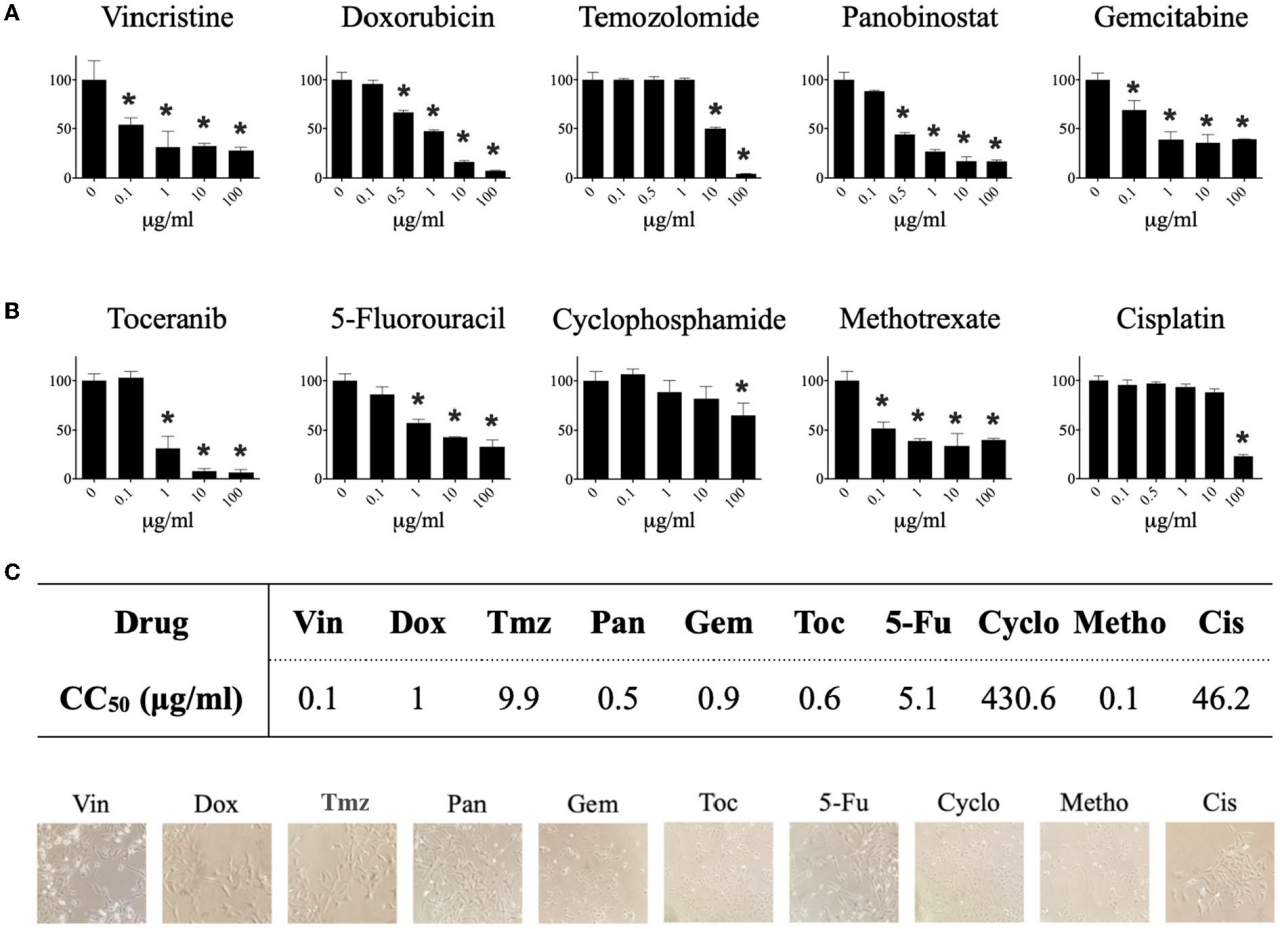
Effect of different pharmacological agents on the viability of the CTVT cell line. CTVT cells (5x10^3^/well) were exposed to different concentrations (from 0 to 100 μg/ml) of the drugs Vin, Dox, Tmz, Pan, Gem, Toc, 5-Fu, Cyclo, Metho, and Cis for 24 h. Relative cell viability was determined by the Alamar-blue assay. **(A)** Each bar graph represents the average of 6 independent measurements ± SD. Statistical significance was performed using Dunnett's *post hoc* test (^*^p < 0.05). **(B)** The CC_50_ of each drug was calculated from the viability data obtained with the Alamar blue assay using the Quest GraphTM ED50 Calculator program (AAT Bioquest, Inc.). The CC_50_ is expressed in μg/ml. **(C)** Representative photographs taken under a bright field microscopy of TVT cells treated with the CC_50_ of each drug. Magnification: 40 ×. Vin, vincristine; Dox, doxorubicin; Tmz, temozolomide; Pan, panobinostat; Gem, gemcitabine; Toc, toceranib; 5-Fu, 5-fluorouracil; Cyclo, cyclophosphamide; Metho, methotrexate; Cis, cisplatin.

Our results show that the tested drugs significantly (^*^ p ≤ 0.05) decreased the viability of CTVT cells in a dose-dependent manner (Figure 1A). The CC_50_ for each drug was determined from the dose–response curve (Figure 1B). The drugs with the highest cytotoxic activity were vincristine (CC_50_ = 0.1 μg/ml), methotrexate (CC_50_ = 0.1 μg/ml), panobinostat (CC_50_ = 0.5 μg/ml), toceranib (CC_50_ = 0.6 μg/ml), gemcitabine (CC_50_ = 0.9 μg/ml), and doxorubicin (CC_50_ = 1 μg/ml). Not all the drugs induced morphological changes in CTVT cells (Figure 1C).

### Effect of single drug treatment on the migration, colony formation, and cell cycle of CTVT cells

**Figure 2:**
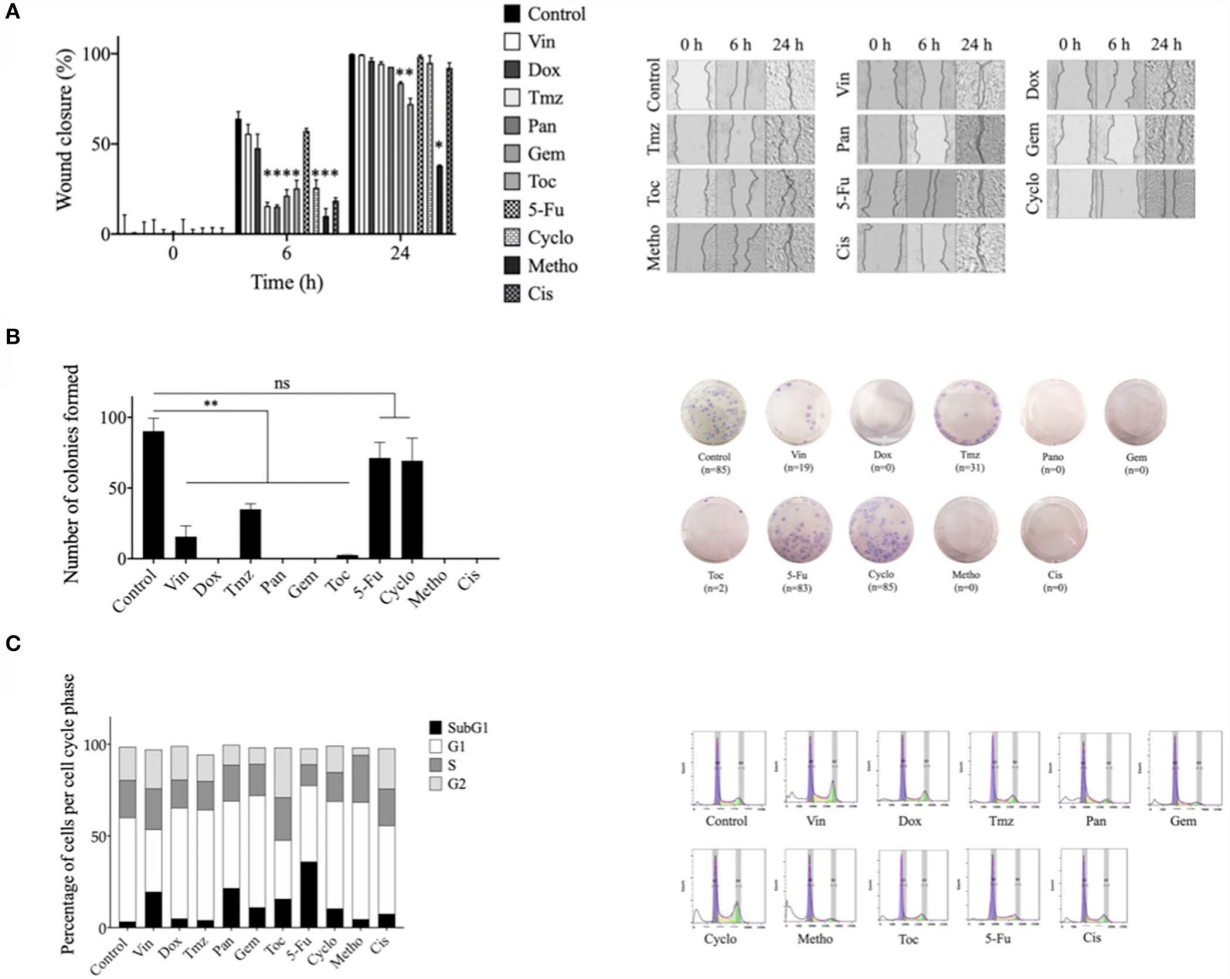
Effect of different pharmacological agents on the migration, colony formation, and cell cycle arrest of the CTVT cell line. **(A)** Wound healing assay with CTVT cells treated with the CC_50_ of Vin, Dox, Tmz, Pan, Gem, Toc, 5-Fu, Cyclo, Metho, and Cis for 24 h. The graph bar represents the wound closure percentage of three replicates ± SD and the image shows representative photographs taken under bright field microscope at a 40 × magnification at the 0, 6, and 24 h time points. **(B)** Colony formation assay of CTVT cells treated with the CC_50_ of Vin, Dox, Tmz, Pan, Gem, Toc, 5-Fu, Cyclo, Metho, and Cis for 24 h, and then incubated with cell culture media for 21 days. The graph bar represents the number of colonies formed by three replicates ± SD and the image shows representative photographs of the colonies formed. **(C)** Cell cycle analysis of CTVT cells treated with the CC_50_ of Vin, Dox, Tmz, Pan, Gem, Toc, 5-Fu, Cyclo, Metho, and Cis for 24 h, as determined by flow cytometry. The graph bar represents the percentage of cells in each cell cycle stage (SubG1, G1, S, and G2), and representative cell cycle distribution histograms are also shown. Statistical significance was performed using Dunnet's *post hoc* test (*p >/=0.05). Vin, vincristine; Dox, doxorubicin; Tmz, temozolamide; Pan, panobinostat; Gem, gemcitabine; Toc, toceranib; 5-Fu, 5-fluorouracil; Cyclo, cyclophosphamide; Metho, methotrexate; Cis, cisplatin.

Temozolomide, panobinostat, gemcitabine, toceranib, cyclophosphamide, methotrexate, and cisplatin significantly (^*^p ≤ 0.05) decreased the migration of CTVT cells for 6 h after treatment removal. Gemcitabine, toceranib, and methotrexate significantly (^*^p ≤ 0.05) decreased the migration of CTVT cells for 24 h after treatment removal (Figure 2A). All tested drugs significantly (^*^p ≤ 0.05) decreased the CTVT cells' capacity to form colonies, except for cyclophosphamide and 5-fluorouracil. Doxorubicin, panobinostat, gemcitabine, methotrexate, and cisplatin completely inhibited the formation of colonies (Figure 2B). Regarding the distribution of cell cycle phases, vincristine (19.1%), panobinostat (21.5%), gemcitabine (11%), toceranib (10.4%), cyclophosphamide (15.7%), and methotrexate (36%), increased the percentage of cells in the subG1 phase as compared to the untreated cells (3.3%). Doxorubicin (15.4%), temozolomide (15.6%), gemcitabine (17.1%), toceranib (15.8%), and methotrexate (11.5%) decreased the percentage of cells in the synthesis phase as compared to the untreated cells (20.4%) (Figure 2C).

### Effect of combined drugs on the viability of CTVT cells

**Figure 3:**
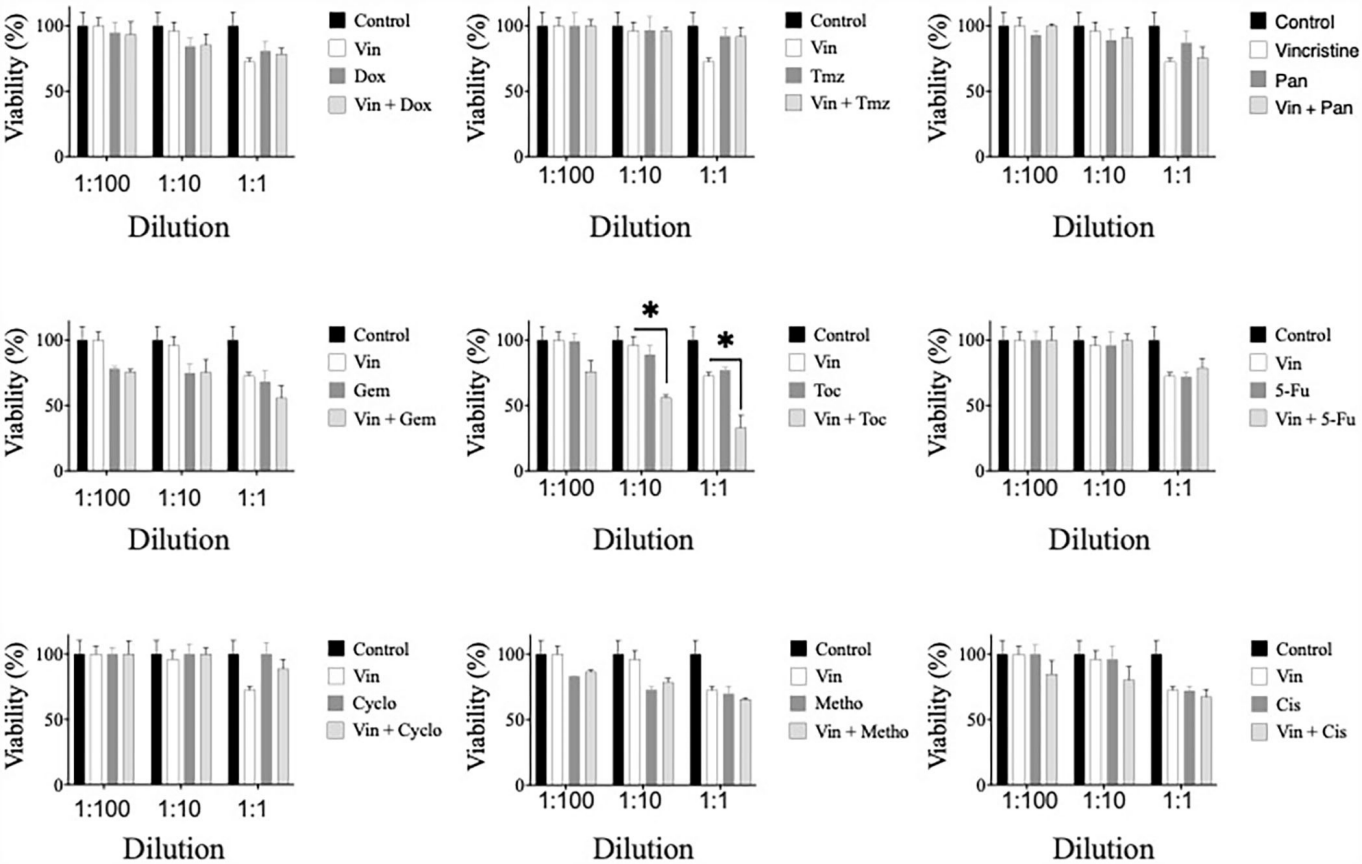
Viability of CTVT cells exposed to the simultaneous administration of vincristine plus different drugs. TVT cells (5x10^3^/well) were exposed to the CC_50_ of Vin plus several dilutions (1:100, 1:10, and 1:1) of the CC_50_ of the drugs Dox, Tmz, Pan, Gem, Toc, 5-Fu, Cyclo, Metho, or Cis for 24 h. The relative cell viability was determined with the Alamar Blue assay. Each bar graph represents the average of 3 independent experiments ± SD. Statistical significance was determined using Tukey's *post hoc* test (*p < 0.05). Vin: vincristine, Dox: doxorubicin, Tmz: temozolomide, Pan: panobinostat, Gem: gemcitabine, Toc, toceranib; 5-Fu, 5-fluorouracil; Cyclo, cyclophosphamide; Metho, methotrexate; Cis, cisplatin.

The potential synergistic effect of vincristine combined with the other drugs was evaluated. For this, CTVT cells were exposed to serial dilutions (1:100, 1:10, and 1:1) of the CC_50_ of vincristine plus the same dilutions of the CC_50_ of each of the other drugs. Only the combination of vincristine plus toceranib in the 1:10 and 1:1 dilution (56 and 33%, respectively), significantly (^*^ *p* ≤ 0.05) decreased the viability of CTVT cells as compared to the effect of the single drugs, 96 and 72.5% for vincristine, and 89 and 77% for toceranib (Figure 3). The CI values were 0.0461 and 0.0068 for the 1:10 and 1:1 dilution, respectively, indicating a synergistic effect. None of the remaining combinations tested exhibited a synergistic effect (Table 1).

**Table 1:** Combination index (CI) values of the effect of different combinations of vincristine and other drugs on the CTVT cells viability.

**Tested drugs**	**Dilution**	**CI**	**Effect**
Vin + Dox	1:1 1:10 1:100	8.896 9.817 16.077	Antagonism Antagonism Antagonism
Vin + Tmz	1:1 1:10 1:100	7.275 2.894 2.894	Antagonism Antagonism Antagonism
Vin + Pan	1:1 1:10 1:100	3.039 5.452 4.659	Antagonism Antagonism Antagonism
Vin + Gem	1:1 1:10 1:100	6.641 1.370 2.781	Antagonism Antagonism Antagonism
Vin + Toc	1:1 1:10 1:100	0.00688 0.04561 5.125	Synergism Synergism Antagonism
Vin + 5-fu	1:1 1:10 1:100	1.734 3.461 3.461	Antagonism Antagonism Antagonism
Vin + Cyclo	1:1 1:10 1:100	Non determined Non determined Non determined	- - -
Vin + Metho	1:1 1:10 1:100	8.376 4.971 3.588	Antagonism Antagonism Antagonism
Vin + Cis	1:1 1:10 1:100	14.7413 4.770 10.167	Antagonism Antagonism Antagonism

Data were analyzed using the Compusyn software. CI <1, =1, and >1, indicate synergism, additive effect, and antagonism, respectively. Vin, vincristine; Dox, doxorubicin; Tmz, temozolamide; Pan, panobinostat; Gem, gemcitabine; Toc, toceranib; 5-Fu, 5-fluorouracil; Cyclo, cyclophosphamide; Metho: methotrexate; Cis, cisplatin.

### Vincristine increases the chemosensitivity of CTVT cells to toceranib

**Figure 4:**
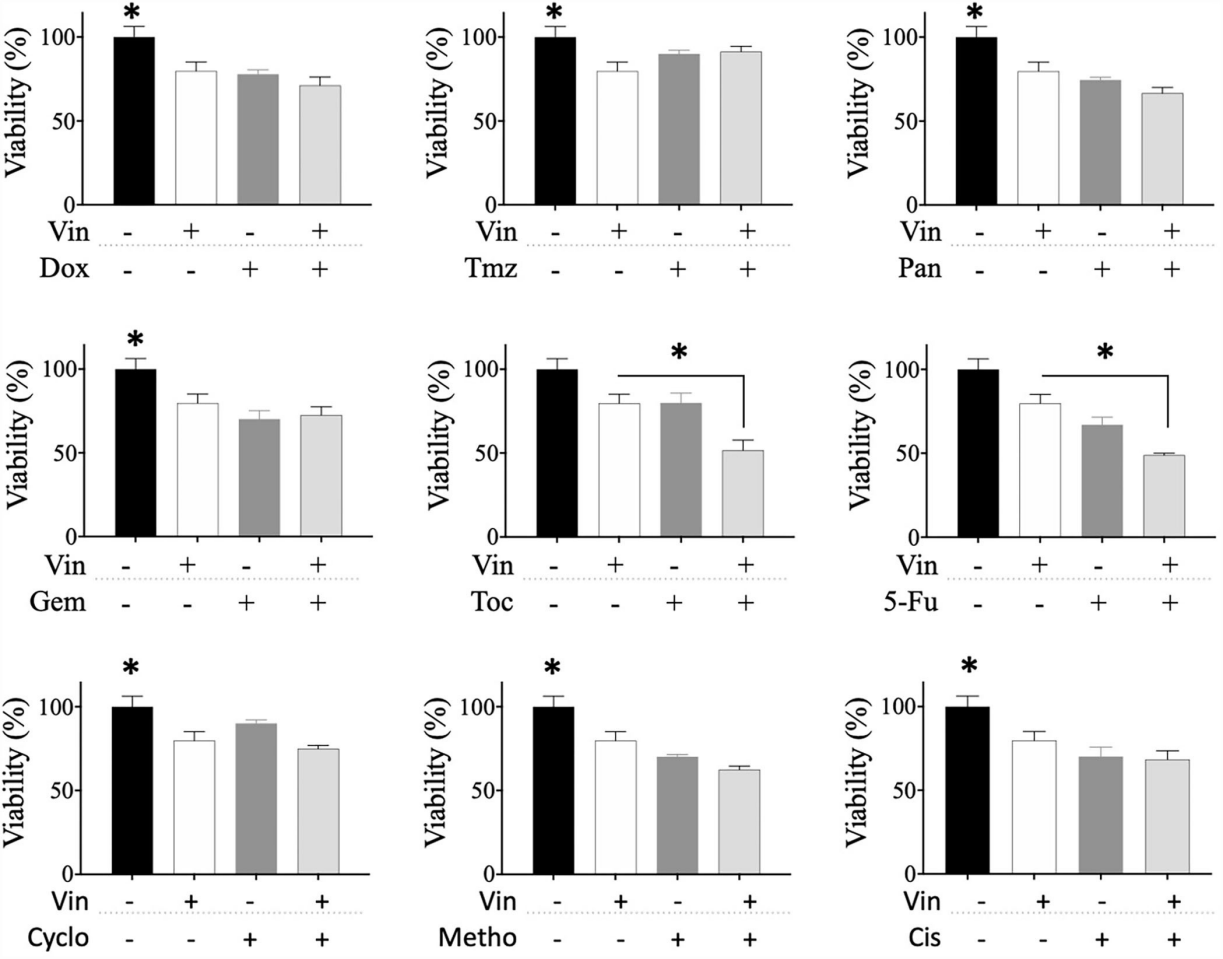
Viability of CTVT cells exposed to the sequential administration of vincristine plus different drugs. CTVT cells (5 × 10^3^/well) were exposed to the CC_50_ of Vin for 24 h. Then Vin was withdrawn, and a 1:1 dilution of the CC_50_ of Dox, Tmz, Pan, Gem, Toc, 5-Fu, Cyclo, Metho, or Cis was added for 24 h. The relative cell viability was determined with the Alamar Blue assay. Each bar graph represents the average of 3 independent experiments ± SD. Statistical significance was determined using Tukey's *post hoc* test (*p < 0.05). Vin, vincristine; Dox, doxorubicin; Tmz, temozolamide; Pan, panobinostat; Gem, gemcitabine; Toc, toceranib; 5-Fu, 5-fluorouracil; Cyclo, cyclophosphamide; Metho, methotrexate; Cis, cisplatin.

**Figure 5:**
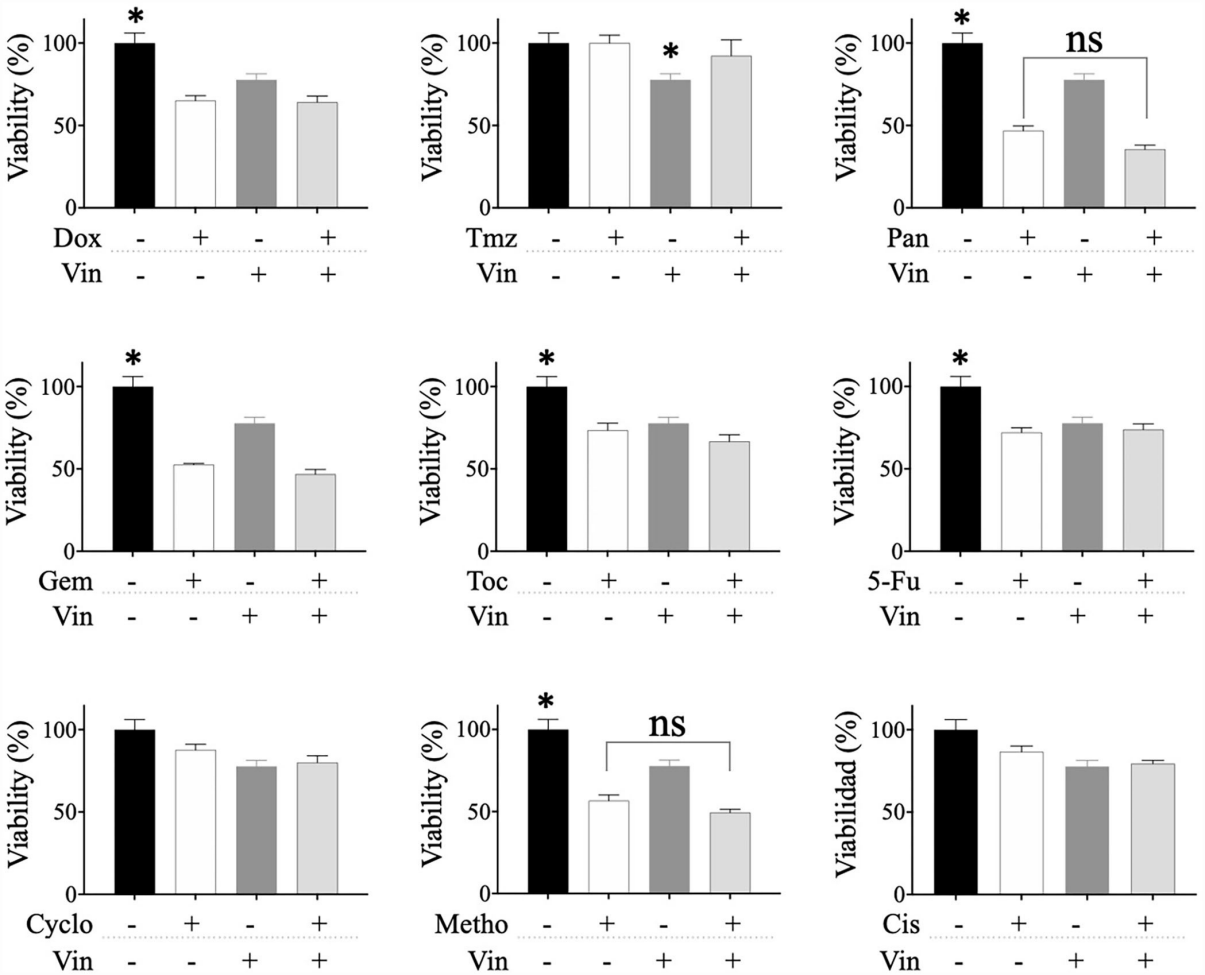
Viability of CTVT cells exposed to the sequential administration of different drugs plus vincristine. CTVT cells (5 × 10^3^/well) were exposed to the CC_50_ of the drugs Dox, Tmz, Pan, Gem, Toc, 5-Fu, Cyclo, Metho, or Cis for 24 h. Then each drug was withdrawn, and a 1:1 dilution of the CC_50_ of Vin was added for 24 h. The relative cell viability was determined with the Alamar Blue assay. Each bar graph represents the average of 3 independent experiments ± SD. Statistical significance was determined using Tukey's *post hoc* test (*p < 0.05), no significance (ns). Vin, vincristine; Dox, doxorubicin; Tmz, temozolomide; Pan, panobinostat; Gem, gemcitabine; Toc, toceranib; 5-Fu, 5-fluorouracil; Cyclo, cyclophosphamide; Metho, methotrexate; Cis, cisplatin.

The effect of sequential treatments on the viability of CTVT cells was also evaluated. Briefly, we treated CTVT cells with the CC_50_ of vincristine for 24 h (sensibilization phase), removed the treatment, and added a 1:1 dilution of the CC_50_ of each of the other drugs. The sequential treatment of vincristine followed by toceranib significantly (^*^ *p* ≤ 0.05) decreased the viability of CTVT cells (48.79%), as compared to each of the vincristine and toceranib alone (79.78 and 67.04%, respectively) (Figure 4). We also evaluated whether different drugs would increase the chemosensitivity of CTVT cells to vincristine; however, our findings revealed that none of the drugs tested increased the chemosensitivity of CTVT cells to vincristine (Figure 5).

## Discussion

In the present study, we investigated the effects of different drugs and their combinations on a CTVT cancer cell line, using the drug sensitivity testing with the aim of proposing new treatment alternatives for CTVT. Drug sensitivity testing is a useful tool that permits to appropriately select a chemotherapeutic of interest (5), easing the process of drug selection by veterinary clinicians for the CTVT treatment. Our results demonstrated that all drugs tested decreased significantly the CTVT cell line cellular viability in a dose-dependent manner, suggesting that each one of these drugs can be used as monotherapy in the treatment of CTVT. This data, however, should be corroborated *in vivo* and used cautiously in cases where vincristine is not available.

The effectivity of each drug has been reported at the clinical level in the treatment of several cancers besides CTVT (15–23). Until now, there were no reports of the use of 5-FU, methotrexate, gemcitabine, temozolomide, panobinostat, and toceranib in CTVT treatment.

These drugs can be used in CTVT treatment due to diverse mechanisms of action: 5-Fluorouracil (5-FU) is an anti-metabolic drug that inhibits the thymidylate synthase and the incorporation of its metabolites into RNA and DNA (24). Methotrexate is an antimetabolite used in cancer treatment, which, by inhibiting the enzyme dihydrofolate reductase, inhibits the synthesis of the purines and pyrimidines that are necessary for nucleic acid synthesis (25). Gemcitabine is a cytidine analog, where two fluorine atoms have replaced the hydroxyl on the ribose. It is a pro-drug and, once transported into the cell, must be phosphorylated by deoxycytidine kinase to an active form. Both gemcitabine diphosphate and gemcitabine triphosphate inhibit processes required for DNA synthesis. After incorporation of gemcitabine nucleotide on the end of the elongating DNA strand, one more deoxynucleotide is added and thereafter, the DNA polymerases are unable to proceed (26). Temozolomide is a monofunctional alkylating agent that induces cytotoxic damage through the creation of methyl adducts at the O6 position on the DNA base guanine. When DNA mismatch repair enzymes attempt to excise the modified nucleotide, they generate single- and double-strand breaks in the DNA that activate apoptotic pathways if no further repairment is available (27–30). Panobinostat is an anticancer agent that acts as histone deacetylase inhibiting tumor cell growth, proliferation, and differentiation, ultimately leading to cell-cycle arrest. As histone acetylation is a fundamental function of panobinostat, it mediates its biological effect through the regulation of gene expression *via* direct histone hyperacetylation and acetylation of non-histone proteins (28). Toceranib phosphate inhibits both normal and mutated tyrosine kinase receptors by competitive inhibition of adenosine triphosphate binding, which is needed for phosphorylation and downstream signaling. The targets of toceranib are split-kinase family elements such as the FMS-like tyrosine kinase-3, KIT, vascular endothelial growth factor receptor 2, and platelet-derived growth factor receptor β (16). These drugs offer different options for mechanisms for cancer cell death at different molecular levels.

On the other hand, gemcitabine, toceranib, and methotrexate were the drugs that showed higher invasive CTVT capacity inhibition. Drugs with the property of inhibiting invasive capacity are associated with the process of cell motility or metastasis and are useful in cancer treatment (31). All the chemotherapeutics evaluated decreased the formation of colonies, but doxorubicin, panobinostat, gemcitabine, methotrexate, and cisplatin completely inhibited it; and cyclophosphamide and 5-fluorouracil don't have the capacity to inhibit the CTVT colony formation. These results are relevant because these drugs offer the possibility to fight against stem cells of CTVT that can generate differentiated cancer clones, inducing recurrence or resistance against treatment, representing a complication in the development of effective therapies (32, 33). When we analyzed the effect of chemotherapeutics on cell cycle progression in CTVT cells, we determined that different drugs act on subG1 phase (vincristine, panobinostat, gemcitabine, toceranib, cyclophosphamide, and methotrexate). It is known that drugs that act on different phases of cell cycle control the cancer cell progression, an important capacity given that some malignancies express gene mutations that participate in cell cycle regulation (34).

Sequential drug administration can maximize therapeutic effects without increasing clinical toxicity (35, 36). To clarify the optimal schedule of vincristine and drug combinations tested in this study, we analyzed the effects of simultaneous and sequential exposure to these combinations. We found that only the combination with vincristine plus toceranib possesses a synergistic effect increasing the chemosensitivity of the CTVT cell line; the other combinations tested do not have an optimal cytotoxic result to be recommended, correlating with clinical studies where the gold standard is the vincristine treatment and its combinatorial use with others chemotherapeutic (35). Combining drugs with capacity to exert antitumor activity through different mechanisms could improve clinical outcomes of the patient with cancer.

The clinical use of vincristine-toceranib as a combined therapy will affect CTVT-bearing dogs as same as *in vitro* test. This combination has been used with one vincristine analog (vinblastine) in mast cell tumor treatment (35). Vincristine is efficient for CTVT treatment, although its mechanisms of action remain under investigation. Vincristine activity has been related to the inhibition of microtubule formation in the mitotic spindle, resulting in an arrest of dividing cells at the metaphase stage. Increased apoptosis, lectin-binding rates, and decreased expression of the antiapoptotic factors, causing in part the CTVT regression (37).

We believed that the combination of vincristine and toceranib increases the antitumor activity because toceranib inhibits both normal and mutated tyrosine kinase receptors that are needed for phosphorylation and downstream signaling, kills tumor cells, and decreases the blood supply to the tumor because of its antiangiogenic effect (16).

Some limitations of this study are that we did not include *in vivo* objectives that demonstrate the efficacy of each drug and combination, and the lack of a vincristine-resistant CTVT cancer cell line to perform drug sensitivity tests and determine the effectivity of the drugs and their combinations. Additionally, the lack of information on CTVT molecular targets at *in silico* level obstructs drug adequation. Furthermore, this study included only some of the chemotherapies used in cancer treatment, and it is necessary to continue testing with more chemotherapeutics, to offer more possibilities for CTVT treatment.

In conclusion, our results confirm the use of vincristine as the gold standard treatment of the CTVT as monotherapy and suggest the use of combinatorial and sequential treatment with toceranib to increase its antitumor effectivity. Furthermore, this study opens the possibility of gemcitabine or methotrexate use as monotherapy due to the vincristine-like chemosensitivity demonstrated. However, these treatment options should be corroborated at the clinical level. Also, it is important to consider determining molecular targets and signaling pathways necessary in the development of CTVT cell lines for better comprehension in the treatment of this disease. The obtained results can, therefore, be adopted into clinical settings with potentially significant influence on CTVT treatment.

## Data availability statement

The original contributions presented in the study are included in the article/supplementary material, further inquiries can be directed to the corresponding authors.

## Author contributions

Conceptualization: MF and SS. Formal analysis: DZ. Funding acquisition and project administration: CR and MF. Investigation: MF, SS, and ES. Methodology: JK, MF, and NP. Supervision: JK and PG. Validation: HP, JK, and NP. Writing—original draft: ES, SS, and PG. Writing—reviewing and editing: YR, MF, SS, and DZ. All authors have read and agreed to the published version of the manuscript.

## Funding

This work was supported by the Laboratorio de Inmunología y Virología of the Universidad Autónoma de Nuevo León, San Nicolás de los Garza, NL, México; by the Fondo Sectorial de Investigación para la Educación, grant A1-S-35951, CONACYT, México, and Laboratorio de Inmunología y Virología, Facultad de Ciencias Biológicas, Universidad autonoma de Nuevo León, San Nicolás de los Garza, NL, México.

## Conflict of interest

The authors declare that the research was conducted in the absence of any commercial or financial relationships that could be construed as a potential conflict of interest.

## Publisher's note

All claims expressed in this article are solely those of the authors and do not necessarily represent those of their affiliated organizations, or those of the publisher, the editors and the reviewers. Any product that may be evaluated in this article, or claim that may be made by its manufacturer, is not guaranteed or endorsed by the publisher.
